# Supplementary material for: Increased aerosol transmission for B.1.1.7 (alpha variant) over lineage A variant of SARS-CoV-2
Source: Res Sq. 2021 Aug 9:rs.3.rs-753550. Preprint. [Version 1] doi: 10.21203/rs.3.rs-753550/v1 (PMC8366800; doi:10.21203/rs.3.rs-753550/v1)
Supplement: Supplement 1 [file 3d3183e3570842ae4dd7cb1b.docx]

**Supplementary Material**

**Increased aerosol transmission for B.1.1.7 (alpha variant) over lineage A variant of SARS-CoV-2**

Julia R. Port*, Claude Kwe Yinda*, Victoria A. Avanzato, Jonathan E. Schulz, Myndi G.Holbrook, Neeltje van Doremalen, Carl Shaia, Robert J. Fischer, Vincent J. Munster#

1. *Laboratory of Virology, Division of Intramural Research, National Institute of Allergy and Infectious Diseases, National Institutes of Health, Hamilton, MT, USA*
2. *Rocky Mountain Veterinary Branch, Division of Intramural Research, National Institute of Allergy and Infectious Diseases, National Institutes of Health, Hamilton, MT, USA*

* These authors contributed equally

# Corresponding author. vincent.munster@nih.gov

**Supplemental Figure 1: Aerosol transmission cages. A/B.** Design of a new caging system in which two hamster cages could be separated at 3 different distances. The distances chosen were nominally 16.5 cm, 106 cm and 200 cm. The distance could be adapted by swapping out a 76 mm inside diameter connection tube. Cages were installed on autoclavable stainless steel shelves (Metro) inside a BSL-4 containment laboratory. Airflow was measured by flowmeters mounted to the shelves. Air was pulled through the system by a negative-pressure pump (Vacuubrand) and filtered through a hepa-filter before the exhaust**.**

**Supplementary Figure 2: B.1.1.7 and lineage A aerosol transmission efficiency at three days post inoculation.** Comparison of aerosol transmission efficiency of lineage A and B.1.1.7 SARS-CoV-2 variants in the Syrian hamster. Donor Syrian hamsters were inoculated with 8x10^4^ TCID_50_ SARS-CoV-2 lineage A or B.1.1.7 variant. After 72 hours, donors were introduced to the upstream cage and sentinels (2:2 ratio) into the downstream cage. Exposure was limited to one hour for B.1.1.7 and lineage A (N = 4, respectively).  **A.** Viral load in oropharyngeal swabs of donors collected 72 hours post inoculation was measured by gRNA and sgRNA. **B/C.** To demonstrate transmission, sentinels were monitored for start and continuation of respiratory shedding. Viral load in oropharyngeal swabs of sentinels was measured by gRNA and sgRNA; swabs were collected at 24, 48 and 72 hours post exposure to the donors. Exposure at 200 cm distance. Truncated violin plots depicting median, quantiles and individuals, blue = lineage A, red = B.1.1.7. Abbreviations: A, lineage A variant; g, genomic; sg, subgenomic.

**Supplementary Figure 3: Dual infection with lineage A and B.1.1.7 variant in the Syrian hamster.** Animals (N = 10) were inoculated with both lineage A and B.1.1.7 variant with 10^2^ TCID_50_ via the intranasal route (1:1 ratio), **A.** Relative weight loss in hamsters after dual inoculation in comparison to lineage A or B.1.1.7 variant inoculation. Graph shows median and 95% CI, N = 10. **B.** Respiratory shedding as measured by sgRNA in oropharyngeal swabs collected at 0, 12, 24, 36, 48, 72 and 120 hours post inoculation. Truncated violin plots depicting median, quantiles and individual, N = 10. **C.** Donor animals (N = 8) were inoculated with both lineage A and B.1.1.7 variant with 10^2^ TCID_50_ via the intranasal route (1:1 ratio), and three groups of sentinels (Sentinels 1, 2 and 3) were exposed subsequently at 16.5 cm distance. Ratio of B.1.1.7 and lineage A variant found in oropharyngeal swabs taken at day 2 post exposure/inoculation for each individual donor and sentinel, measured by duplex-qRT-PCR and depicted by ct foldchange (B.1.1.7 over lineage A variant). Colors refer to scale on the right. Samples for which only one variant was detected by PCR were set to 25,000. Abbreviations: A, lineage A variant; sg, subgenomic.

**Supplementary Table 1: Aerosol Transmission Cage Validation Parameters.** Special transmission cages were designed to model airborne transmission between Syrian hamsters. Volume of air (cages plus connection tube), air flow velocity in the tube and time for particles to traverse is provided at a cage air change rate of 30/h.

| Cage system | Volume (L) | Linear velocity (cm/min) | Time (sec) |
| --- | --- | --- | --- |
| Connecting tube (200 cm) | 1073 | 420 | 26.7692784 |
| Connecting tube (106 cm) | 945 | 370 | 15.059599 |
| Connecting tube (16.5 cm) | 837 | 327 | 2.80215197 |

**Supplementary Table 2: anti-spike ELISA results for sentinels in aerosol transmission studies.** Presence of SARS-CoV-2 spike IgG antibodies in sentinels co-housed at 16.5, 106 and 200 cm distance from lineage A or B.1.1.7(*) variant inoculated donor hamsters. Detected in serum obtained 14 days post exposure. negative (neg): optical density (at 450 nm) < 0.124, positive (pos) optical density (at 450 nm) ≥ 0.124.

| Condition | Animal | Seroconverted |
| --- | --- | --- |
| 16.5 cm | S1 | pos |
|  | S2 | pos |
|  | S3 | pos |
|  | S4 | pos |
| 106 cm | S1 | pos* |
|  | S2 | pos* |
|  | S3 | pos* |
|  | S4 | pos* |
|  | S5 | pos |
|  | S6 | pos |
|  | S7 | pos |
|  | S8 | pos |
| 200 cm | S1 | pos* |
|  | S2 | pos* |
|  | S3 | pos* |
|  | S4 | pos* |
|  | S5 | pos |
|  | S6 | pos |
|  | S7 | pos |
|  | S8 | pos |

**Supplementary Table 3: Pathological assessment of lungs collected at 5 days post exposure.** Donor animals (N = 8) were inoculated with both lineage A and B.1.1.7 variant with 10^2^ TCID_50_ via the intranasal route (1:1 ratio), and three groups of sentinels (Sentinels 1, 2 and 3) were exposed subsequently at 16.5 cm distance. At five days post exposure, lungs were observed for gross pathology.

| Identification | COVID-19 phenotype | Gross pathological assessment |
| --- | --- | --- |
| Sentinel 1.1 | N/A | bilateral focally extensive dorsal poorly circumscribed dark red discoloration |
| Sentinel 1.2 | N/A | multifocal poorly circumscribed dark red discoloration |
| Sentinel 1.3 | Yes | multifocal to coalescing foci of well circumscribed dark red discoloration suggestive of interstitial pneumonia |
| Sentinel 1.4 | Yes | multifocal foci of well circumscribed dark red discoloration suggestive of interstitial pneumonia |
| Sentinel 1.5 | N/A | focus of discoloration on the ventral margin on the left lobe, but no clear foci |
| Sentinel 1.6 | Yes | multifocal foci of well circumscribed dark red discoloration suggestive of interstitial pneumonia |
| Sentinel 1.7 | Yes | multifocal to coalescing foci of well circumscribed dark red discoloration suggestive of interstitial pneumonia |
| Sentinel 1.8 | Yes | multifocal foci of well circumscribed dark red discoloration suggestive of interstitial pneumonia |
| Sentinel 2.1 | N/A | multifocal foci of poorly circumscribed dark red discoloration suggestive of interstitial pneumonia |
| Sentinel 2.2 | N/A | focally extensive foci of poorly circumscribed dark red discoloration |
| Sentinel 2.3 | No | normal |
| Sentinel 2.4 | No | normal |
| Sentinel 2.5 | No | normal |
| Sentinel 2.6 | No | normal |
| Sentinel 2.7 | N/A | focally extensive foci of poorly circumscribed dark red discoloration |
| Sentinel 2.8 | N/A | focally extensive foci of poorly circumscribed dark red discoloration |
| Sentinel 3.1 | No | normal |
| Sentinel 3.2 | No | normal |
| Sentinel 3.3 | No | normal |
| Sentinel 3.4 | No | normal |
| Sentinel 3.5 | No | normal |
| Sentinel 3.6 | No | normal |
| Sentinel 3.7 | No | normal |
| Sentinel 3.8 | No | normal |

**Supplementary Table 4: qRT-PCR and sequencing results for donor and sentinel animals.** Donor animals (N = 8) were inoculated with both lineage A and B.1.1.7 variant with 10^2^ TCID_50_ via the intranasal route (1:1 ratio), and three groups of sentinels (Sentinels 1, 2 and 3) were exposed subsequently at 16.5 cm distance. Viral load in copies/reaction (measured by qRT-PCR) and percentage of B.1.1.7 detected in oropharyngeal swabs taken at day 2 post exposure for each individual donor and sentinel, determined by deep sequencing (expressed as %) and expressed as fold-change over lineage A as measured by duplex-qRT-PCR.

| Animal | raw reads | qc reads | Viral RNA copies/reaction | % B.1.1.7 | PCR fold-change |
| --- | --- | --- | --- | --- | --- |
| Donor 1 | 9697 | 9289 | 1239 | 54 | 0.87408891 |
| Donor 2 | 77807 | 75255 | 85054 | 45 | 0.67126948 |
| Donor 3 | 78379 | 75218 | 103141 | 67.66667 | 1.69959284 |
| Donor 4 | 87992 | 85444 | 101401 | 70 | 2.1594464 |
| Donor 5 | 85062 | 82728 | 199462 | 74.66667 | 2.93509401 |
| Donor 6 | 135883 | 132657 | 254909 | 62 | 1.48700317 |
| Donor 7 | 45156 | 43638 | 71343 | 57.66667 | 1.2711726 |
| Donor 8 | 93687 | 91017 | 93356 | 57.66667 | 1.30263123 |
| Sentinel 1.1 | 65408 | 63416 | 126315 | 99 | 4161.61905 |
| Sentinel 1.2 | 41250 | 39943 | 38272 | 47 | 1.18602528 |
| Sentinel 1.3 | 112221 | 107500 | 20842 | 98.33333 | 395.151923 |
| Sentinel 1.4 | 107062 | 101891 | 62637 | 96 | 110.145084 |
| Sentinel 1.5 | 91154 | 88209 | 42500 | 96.66667 | 803.405938 |
| Sentinel 1.6 | 136060 | 131881 | 16994 | 58 | 1.12334412 |
| Sentinel 1.7 | 44594 | 43114 | 115352 | 98 | 2303.5488 |
| Sentinel 1.8 | 99311 | 96261 | 169096 | 16 | 0.12975316 |
| Sentinel 2.1 | 131545 | 126930 | 29513 | failed | 0.00073727 |
| Sentinel 2.3 | 33284 | 31942 | 4003 | 100 | 18001.5885 |
| Sentinel 2.4 | 25906 | 25000 | 4056 | 100 | 20922.7397 |
| Sentinel 2.6 | 10848 | 10219 | 522 | failed | * only B.1.1.7 detected |
| Sentinel 3.2 | 154787 | 141295 | 65 | failed | * only B.1.1.7 detected |

**Supplementary Table 5: Duplex-qRT-PCR Primers and Probes**

| Primer/probe | Sequence (5’🡪 3’) |
| --- | --- |
| VM3256-RML-(drop out) | VIC-TGTTACTTGGTTCCATGCTATACATG-ZEN-IBHQ |
| VM3256-RML-(detection) | FAM-GTTCCATGCTATCTCTGGGACC--ZEN-IBHQ |
| VM3254-RML-F | AAAGTTTTCAGATCCTCAG |
| VM3255-RML-R | GTTAGACTTCTCAGTGGAAG |

**Supplementary Table 6: Sequence results of virus stock B.1.1.7**

| **ORF** | **a.a. change** | **Percentage** |
| --- | --- | --- |
| nsp6 | D165G | 14 |
| nsp6 | L257F | 18 |
| nsp7 | V11I | 13 |
